# The validity of open-source data when assessing jail suicides

**DOI:** 10.1186/s40352-018-0069-2

**Published:** 2018-05-09

**Authors:** Amanda L. Thomas, Jacqueline Scott, Jeff Mellow

**Affiliations:** John Jay College of Criminal Justice, 524 W 59th St., Haaren Hall, Rm. 631, New York, NY 10019 USA

**Keywords:** Jail suicide, Open-source data, Official data, DCRP

## Abstract

**Background:**

The Bureau of Justice Statistics’ Deaths in Custody Reporting Program is the primary source for jail suicide research, though the data is restricted from general dissemination. This study is the first to examine whether jail suicide data obtained from publicly available sources can help inform our understanding of this serious public health problem.

**Methods:**

Of the 304 suicides that were reported through the DCRP in 2009, roughly 56 percent (*N* = 170) of those suicides were identified through the open-source search protocol. Each of the sources was assessed based on how much information was collected on the incident and the types of variables available. A descriptive analysis was then conducted on the variables that were present in both data sources. The four variables present in each data source were: (1) demographic characteristics of the victim, (2) the location of occurrence within the facility, (3) the location of occurrence by state, and (4) the size of the facility.

**Results:**

Findings demonstrate that the prevalence and correlates of jail suicides are extremely similar in both open-source and official data. However, for almost every variable measured, open-source data captured as much information as official data did, if not more. Further, variables not found in official data were identified in the open-source database, thus allowing researchers to have a more nuanced understanding of the situational characteristics of the event.

**Conclusions:**

This research provides support for the argument in favor of including open-source data in jail suicide research as it illustrates how open-source data can be used to provide additional information not originally found in official data. In sum, this research is vital in terms of possible suicide prevention, which may be directly linked to being able to manipulate environmental factors.

**Electronic supplementary material:**

The online version of this article (10.1186/s40352-018-0069-2) contains supplementary material, which is available to authorized users.

## Background

Approximately 12 million individuals cycle through U.S. local jails each year with an estimated 721,300 daily jail confinements (Bureau of Justice Statistics 2016; Subramanian et al. 2015). Research into this population demonstrates that jail inmates have a much higher rate of mental and substance abuse disorders than that found in state and federal prisoners. It is estimated, for example, that more than 64% of jail inmates have a mental health problem, compared to 56 and 45%, respectively, of state and federal prisoners (Bureau of Justice Statistics 2006). It is also important to note that statistics on jail inmates may be grossly underestimated due to the high turnover rate of the general jail population throughout the year (Tartaro and Ruddell 2006).

Though suicide is not in itself a mental illness, it may often be the result of undiagnosed or untreated mental health disorders (Baillargeon et al. 2009; Hanson 2010; He et al. 2001; Novick and Remmlinger 1978). Jails are operating as de facto mental health facilities without adequate behavioral health resources and are experiencing growing suicide rates (Hanson 2010). Suicide is the leading cause of death in local jails, accounting for more than 30% of inmate deaths (Goss et al. 2002; Hayes 1997; Noonan 2016; Noonan et al. 2015). By 2013 the rate of jail suicides (46 per 100,000 inmates) was higher than suicide rates in state prison populations (15 per 100,000) and in the community (13 per 100,000) (Noonan et al. 2015). Even within jails, suicide rates vary drastically by size, with smaller jails (e.g., those with less than 100 beds), reporting a suicide rate roughly five times higher than the larger jails (Tartaro and Ruddell 2006).

The epidemic proportion of suicides in local jails has sparked an increased interest in researching the correlates of jail suicides and analyzing why jails, unlike prisons, pose such a high suicide risk to its population (Bonner 2000; Dillon 2013). Studies have identified several suicide risk factors including the lack of suicidal ideation screening protocols and the psychological and environmental distress of being incarcerated for the first time (Bonner 2000; Hanson 2010; Hayes 2012, 2013; Schaefer et al. 2016; Tartaro and Ruddell 2006). What these various studies have in common is their reliance on administrative data from federal prisons, state prisons, and local jails, specifically data from few primary sources: the US Bureau of Justice Statistics Deaths in Custody Reporting Program (DCRP) (see Hanson 2010; Hayes 2013) and state- and/or local-sources (see Dillon 2013; Goss et al. 2002; Winter 2003).

Relying solely on administrative data, in of itself, is not necessarily problematic. However, there are several important challenges which present themselves when using this data for research, including missing data, reporting/recording problems, limited access to data, the time lag between data collection and release, and the limited number of variables collected (see Hampton 2016; Zeng et al. 2016). In 2016, Zeng and colleagues assessed some of these concerns by comparing the data from the DCRP and the National Death Index (NDI) from 2007 through 2010. Their research identified several discrepancies related to administrative data collection (Zeng et al. 2016, p. 1); for example, a significant anomaly occurred in 2008 when more than 20% of DCRP data was missing in the cause of death field for that year (Zeng et al. 2016). Another study of official records of in-custody deaths in Oklahoma found multiple inconsistencies between the number of deaths reported via federal and state records (Hampton 2016). A closer review of the data noted that the discrepancies in the number of inmates who died in custody, as they were reported to state and federal agencies, were found to have occurred in 2011, 2013, and 2014 in Cleveland County Jail (Hampton 2016). Despite the fact that there is a difference between state and federal systems, there should be no discrepancies found in the number of “deaths in custody” that are reported to either agency. Hampton’s (2016) research concluded that this difference might be a result of how a death in custody is defined and with who is tasked with the responsibility of submitting these reports.

While there has been a continued effort in the field of corrections to critically examine the phenomenon of jail suicides, researchers have yet to incorporate open-source databases in their analyses. Also referred to as publicly available data, open-source search protocols have been frequently utilized to develop datasets to study a diverse range of social science phenomena (see Ackerman and Pinson 2016; Chandra et al. 2014; Gruenwald et al. 2013), particularly those surrounding controversial social issues (Ackerman and Pinson 2016). However, it is critical that researchers building open-source datasets explicitly engage in a rigorous evaluation process of both the source(s) and information gathered (Ackerman and Pinson 2016). Furthermore, these researchers also posit that transparency is critical and “that any procedures should be … explicitly coded into the database itself” (Ackerman and Pinson 2016, p. 623).

Most of the published research on jail suicides in the last two decades utilizes administrative data collected by individual facilities that are then submitted to the US Bureau of Justice Statistics Deaths in Custody Reporting Program (DCRP) (Hanson 2010; Hayes 2013). The DCRP was established following the passage of the Death in Custody Reporting Act of 2000 (Public Law 106–297) and collects data annually on inmates’ deaths from roughly 2900 local jails (Kaeble and Glaze 2016). By law, the Act requires the individual state to furnish the federal government on a quarterly basis with “information regarding the death of any person who is in the process of arrest, is en route to be incarcerated, or is incarcerated at a municipal or county jail, State prison, or other local or State correctional facility (including any juvenile facility)” (Legal Institute Information n.d.). To date, it is the only federal government survey to collect annual data on jail deaths, including jail suicides.

According to the Act, states are only required to collect the following individual-level suicide information: “(A) the name, gender, race, ethnicity, and age of the deceased; (B) the date, time, and location of death; and (C) a brief description of the circumstances surrounding the death” (Legal Institute Information n.d.). In compliance with the Act, the Bureau of Justice Statistics developed the Death in Custody Quarterly Report on Inmates Under Jail Jurisdiction in 2000 (Kaeble and Glaze 2016). In addition to the required data points, this 19-item survey also collects individual information (i.e., inmate confined at any time to a mental health unit or facility since admissions), criminal justice characteristics of the deceased (i.e., length of stay prior to death; convicted, probation/parole violator, or detainees’ legal status; and criminal offense type) and detailed circumstances of the death (i.e., death location, medical examiner verification of cause of death, cause of suicide death, and time of death) (Form CJ-9 n.d.). Seven different death locations are listed in the survey (e.g., general housing, segregation housing) with the option of writing in a location. The cause of deaths question gives three examples (i.e., hanging, knife/cutting instrument, intentional drug overdose) and requires a write-in response (Form CJ-9 n.d.).

Despite the fact that the DCRP has been responsible for collecting data on inmate deaths since 2000, questions still exist regarding the validity and reliability of this program, particularly because DCRP forms are completed and submitted by a wide range of correctional professionals (Zeng et al. 2016). To better understand this issue, Zeng et al. (2016) compared the cause of death findings reported via the DCRP and the National Death Index (NDI) for 2007 through 2010. This research found that the DCRP had less missing data relating to the cause of death (1.9%) compared to the NDI (6.3%). However, within the DCRP there was more missing data on the cause of death in jails (6.3%) than in state prisons (0.7%). The DCRP also reported slightly higher rates of jail suicide deaths (31%) between 2007 and 2010 than the NDI (29.6%). Zeng et al. (2016) recommends incorporating both the NDI with the DCRP in order to obtain “more accurate and comprehensive COD data” (p. 13).

Recognizing the methodological limitation of the DCRP data, several researchers (e.g., Hayes 2013; Tartaro and Ruddell 2006; Winter 2003) supplement the DCRP data with a secondary survey to the facilities concerned to follow up with a more detailed review of the critical incident. For example, in Winter’s (2003) study, the follow-up questionnaire consisted of more in-depth questions surrounding the inmate’s demographic characteristics, arrest history and current charges, as well as questions that assessed the inmate’s physical and mental health prior to the incident. Other studies have collected jail suicide data at the state- (e.g., Winter 2003; Woog 2016) or local-level (e.g., Goss et al. 2002; Woog 2016). Woog (2016), for example, analyzed suicides in Texas jails using Texas Commission on Jail Standards’ county jail death data. Winter (2003) identified the suicides that occurred within local jail facilities via administrative records obtained from the State Office of Detention Facilities for one Midwestern state, then followed up with a secondary questionnaire in order to obtain the most accurate information available. Goss et al.’s (2002) study used members of the King County, Washington jail psychiatric staff to collect all pertinent data surrounding jail suicides.

Despite their differences, all of the published sources reviewed attempt to examine suicides in correctional facilities by identifying the individual-, situational-, and facility-level risk factors for jail suicides, and the majority of research conducted in this field focused on studying this phenomenon quantitatively.^1^ As such, the following section reviews the empirical literature on jail suicides by characteristic type as they relate directly to this study, focusing specifically on individual-level and facility-level characteristics of jail suicides.

### Individual-level characteristics of jail suicides

Hayes’ (2010) review of 464 jail suicides found that individuals who were described as single, white men who were around the age of 35 experienced the highest rates of jail suicides. Furthermore, approximately one-third of the inmates who committed suicide had a history of mental illness (38%) and [or] a history of suicidal behavior (34%). In this study, 24% of jail suicides occur within the first 24 h of arrest [or intake] with another 27% from day two to 2 weeks, and suicides were more likely to occur around the times when inmates were expected to appear for court proceedings (Hayes 2010).

Subsequent to Hayes’ (2010) seminal piece on correctional suicide, Hanson (2010) specifically focused on examining “clean” jail suicides. A “clean” suicide is one that is committed by an individual who has “no prior psychiatric history” (Hanson 2010, p. 7). Hanson (2010) argued that individual characteristics (e.g., experiencing more life stressors and relationship troubles) and environmental factors (e.g., violent behavior within the institution, institutional overpopulation, and institutional instability) might be directly related to the rate of suicide in jail facilities. His research found that some inmates who commit suicide escape detection during the intake process because they have never experienced (or reported) a mental illness (or episode) prior to incarceration (Hanson 2010). As such, these individuals prove to be especially difficult to identify during intake.

The majority of state and local-level jail suicide studies (see Dillon 2013; Goss et al. 2002; Winter 2003) are consistent with Hayes’ (2010), Hayes’ (2013) and Hanson’s (2010) findings that jail suicide victims are predominantly white males, with an average age of 30, and currently detained for a violent felony charge with a history of previous arrests. Furthermore, the research consistently finds that inmates who commit suicide are more likely to be single-celled and kill themselves within the first month of their detention (Dillon 2013; Goss et al. 2002; Winter 2003).

### Facility-level characteristics of jail suicides

The role of facility-level characteristics also plays a role in jail suicides. Research suggests that the extremes of jail housing, overcrowding and single-cell or isolated housing, is a critical factor in jail suicides (Dye 2010). While single-cell occupancy may appear to be beneficial in a jail setting, research has shown that there are also significant, unintended drawbacks (Reeves and Tamburello 2014). The DCRP data reports that the majority (80%) of correctional suicides occur in the victim’s cell (Mumola 2005). Bonner’s (2000) meta-analysis of prison and jail suicides identified single-cell housing as a “common denominator” among the research in explaining successful suicide attempts. Villarreal (2015) also found that suicides are more likely to occur when an inmate is not supervised and remains alone. Surprisingly, most correctional suicide studies do not attempt to systematically uncover the “types of stresses that predispose toward or trigger carceral suicides” (Felthous 2011, p. 1550), though Bonner (2000) notes that correctional suicides are correlated with the levels of hopelessness and depression experienced by inmates housed in single cells.

Correctional overcrowding is another factor found to be correlated with correctional suicides (Ciuhodaru et al. 2009; Dillon 2013; Jewkes 2011; Tartaro and Ruddell 2006; Villarreal 2015). Hanson (2010) found that institutional overcrowding combined with institutional instability and the presence of a violent setting may also lead to an increase in correctional suicides. In further support, Villarreal (2015) also found that suicides are more likely to occur in maximum-security facilities that are over-crowded.

The size of the correctional facility is another environmental factor that has been linked to jail suicides (Dillon 2013; Tartaro and Ruddell 2006). Tartaro and Ruddell’s (2006) research found that small-medium sized facilities (e.g., those with less than 100 beds) were plagued with a suicide rate two to five times higher than larger correctional facilities, with facilities with an average daily population of less than 50 having the highest suicide rates. While, Dillon’s (2013) research suggests that the suicides rate for smaller facilities is actually six times higher than the suicide rate for larger correctional facilities. Tartaro and Ruddell (2006) determined that less than one-fifth (approximately 355) of the small-medium facilities in their study failed to utilize formal suicide assessment during initial inmate intake, “and only slightly more than one-half of these facilities provided annual suicide training to jail officers” (p. 81).

Goss et al.’s (2002) research suggests that making strategic decisions about structural changes to the actual facility may help decrease suicides in correctional facilities (e.g., using Plexiglas barriers to prevent jumping). Hayes (2013) also strongly supports the idea of utilizing suicide-resistant architecture (e.g., fixtures that are tamper-proof and fiberglass bunks) and “anti-suicide” products (e.g., safety smocks). Additionally, Villarreal (2015) suggests that penitentiary designers should aim to reduce “over-institutionalization” of the facility and remove any aspect that may be used to facilitate self-harm.

All of this previous research suggests that researchers are still trying uncover and understand all of the possible correlates that may be associated with jail suicides (Dillon, 2013; Goss et al. 2002; Hanson 2010; Hayes 2010, 2012, 2013; Tartaro and Ruddell 2006; Villarreal 2015; Winter 2003). Thus, by creating a database that contains ample event descriptors, researchers can begin to explore different individual and situational factors that may decrease the likelihood of jail suicides. In sum, this study is crucial to the field, in that it may potentially present researchers and practitioners with an alternative outlet to consult for a more robust explanation as to which correlates are associated with jail suicides. As such, this study aims to determine the validity and reliability of utilizing open-source jail suicide data to improve understanding on the individual-, incident-, and facility-level characteristics of jails suicides. Specifically, the goal of this research is to determine if using an open-source protocol can replicate the Bureau of Justice Statistics’ jail suicide national prevalence rates, and add to a more comprehensive understanding of jail suicide at a national level.

## Methods

### The current study

This study aims to add to the existing literature surrounding jail suicides by examining the validity and reliability of utilizing open-source jail suicide data in conjunction with official jail suicide reports. There are two main goals of conducting this type of research. The first is to evaluate how the newly synthesized data can be used to better inform the development of new correctional policies (e.g., implementing the use of “anti-suicide” products and/or utilizing suicide-resistant architecture) surrounding jail suicides. The second is to increase our understanding of the correlates of jail suicide.

The present study is a retrospective, descriptive study that examines whether open-source data can be used in conjunction with official data to create a more robust dataset on jail suicides. This was assessed by analyzing the types of data presented in official Bureau of Justice Statistics’ reports compared with data gathered via an open-source protocol. This study used a sample of jail suicides occurring in local correctional facilities within the United States from January 1, 2009 to December 31, 2009. Of the 304 suicides that were officially reported through the DCRP, 170 (almost 56%) suicides were identified through the open-source search protocol.

In this study, local correctional facilities were defined as any city, municipal or county detention facility that was responsible for detaining an individual either after an arrest has been made or while they await sentencing and/or trial (Bonner 2000). Here, the unit of analysis (i.e., jail suicides) was an event/incident that occurred at the facility level. Furthermore, each of these incidents were comparatively examined (e.g., official data and open-source data) via four modalities: (1) the demographics of the victim, (2) the location of the occurrence within the facility, (3) the location of the occurrence by state, and (4) the size of the facility. Lastly, in regards to terminology, the term validity refers to the accuracy of a measurement; whereas, reliably refers to measurement consistency (Maxfield and Babbie 2015).

### Data sources

The data used in this study was secondary data from two main sources – official and open-source. The Deaths in Custody Reporting Program (DCRP) (discussed above) supplied the official data, which was publicly available from the US Bureau of Justice Statistics’ webpage (see https://www.bjs.gov/index.cfm?ty=tp&tid=1). The open-source suicide data was obtained from the Jail Correctional Incident Database (JCID). The JCID is a database of critical incidents (e.g., escapes, suicides, riots) that have occurred in U.S. local jails, from 2009 on (Peterson et al. 2016). This database was originally developed to gather detailed information on inmate escapes, but was then expanded to include information on other violent incidents (i.e., suicides and riots). Furthermore, the original research team that was involved in sourcing this database was comprised of Professor Jeff Mellow and eight volunteer research assistants. Therefore, as part of an ongoing project, this study was approved as exempt from needing ethics approval by the Human Research Protections Program at John Jay College of Criminal Justice.

The JCID used a multi-state, open-source, internet-based search protocol to identify and code information on jail suicides. A number of strategies were used to effectively and efficiently source and search for jail suicides online. First, a custom date range of January 1, 2009 through January 31, 2010 was included for all searches to ensure that jails suicides committed at the end of December 2009 had time to be identified and reported in the media. Secondly, jail suicides were searched using Boolean search keywords (e.g., “jail”, “detention”, “inmate”, “killed”, “die*”, “suicid*”, “hang*”, “hung”) and Boolean operators (“and”, “or”, “not”, “*”). A search for “suicid*”, for example, returns the terms “suicide” and “suicidal” while “hang*” also includes “hanged.” Finally, jail suicides were searched by individual states as a way to filter a large number of search results to review at a time. Suicide incidents were collected based on the following criteria: (1) the suicide was a confirmed inmate suicide (not an attempted suicide), (2) the suicide occurred in a local U.S. jail, (3) the suicide occurred in 2009, and (4) the information was sourced online using Google Search, Google News, and *Corrections.com*, a national serial correctional news archive.

Once the incidents were collected, each identified incident was assigned to a specific researcher who systematically searched the incident in online search engines (Microsoft, Firefox, and Google) to uncover all publicly available materials on it. In this way, each incident was treated as a case study with the goal of compiling as much open-source information as possible. Additional jail suicide cases uncovered during the second stage were treated as separate incidents and added to the database. Finally, the sourced information was coded into a relational database according to variables identified by the research team from the literature. These included facility- (18 variables), incident- (22 variables), and suicide victim- (24 variables) level variables (see Additional file 1). Additionally, facility-level data (e.g., jail size and daily rated capacity) was also gathered from the American Correctional Association’s National Jail and Adult Detention Directory (2012), which were supplemented where necessary by jail and county webpages. These variables were coded into a testable form for future analysis.

### Methodological procedures

#### Combining and comparing variables

While both data sources provided critical information pertaining to jail suicides, they often diverged on how much information was collected and the types of variables that were available (see Table 1).

**Table 1:** Type of Variables Collected by Each Source

Variable Type	BJS		JCID	
	N	%	N	%
Individual-level	7	36.8	24	37.5
Facility-level	1	5.3	18	28.12
Incident-level	10	52.6	22	34.38
Other	1	5.3	0	0
Total	19	100	64	100

The most striking difference was found in the level of detail included in the open-source database as compared to official data. For example, the open-source database had significantly more information on the facility (e.g., the year it was built, its capacity, the percent over capacity, and annual admission) and on the incident (e.g., the day of the week that it occurred on, the suicide type, how the suicide was completed and with what, and the exact location within the facility that it occurred). Furthermore, official data reports jail suicide data aggregately, making the examination of any single year or variable difficult. Despite these differences in individual-, facility-, and incident-level variables, there were four variables that were consistent in both data sources, which included demographics, location within the facility, location by state, and facility size.

Regardless of this overlap, not all variables were classified the same (see Additional file 1). For example, the JCID provided the exact age of the victim, whereas the official data (DCRP) provided the ages of suicides victims in ranges. As such, the JCID data had to be collapsed to fit the DCRP data classifications. Furthermore, the JCID referred to inmate conviction status as “sentenced vs. detained,” whereas DCRP data classified legal status as “convicted or unconvicted.” Thus, the JCID data had to be reclassified appropriately to conduct the analyses. Specifically, “sentenced” was reclassified as “convicted” and “detained” was reclassified as “unconvicted.”

There were also differences in the classification of the location where the suicide occurred. The JCID provided more detailed information on this variable (18 different classifications), whereas DCRP data only had 7 classifications. Again, in order to compare the data, the open-source JCID categories were collapsed to match the DCRPs categories of: general housing, segregation unit, medical unit, mental health unit, in transit, or elsewhere/outside. Lastly, we also included one additional classification utilized by JCID – missing.

Finally, there were some issues with comparing the facility size using official data and open source data. Here, DCRP data reports the amount of suicides that occurred in facilities by their size (e.g., the smallest facilities have less than 50 beds, then there are facilities that have 50 to 99 beds, 100 to 149 bed) for only 1 year (i.e., 2002). However, the JCID does not provide that information. Instead, information pertaining to facility size (i.e., average daily population and rated capacity) was located through the American Correctional Association’s National Jail and Adult Detention Directory (2012). Despite these inconsistencies, the data was still compared to determine whether there was a significant change in where jail suicides occurred based on facility size.

## Results

Of the 304 jail suicides reported in the DCRP for 2009, 170 of them (almost 56%) were located in the JCID. With the 170 matching results, descriptive analyses were conducted on the two sources of data.

Official demographic data for local jail suicides is not publicly available, likely for reasons of confidentiality; therefore, aggregate data from 2000 to 2014 (N: 4508) was compared with 2009 JCID data (N: 170). However, despite these diverging date ranges and differences in overall counts, the demographic makeup of the deceased in jail suicides were nearly identical between the two data sources, with an overall correlation of 0.99. As shown in Table 2, in both data sources, 91% of the deceased were male and 9% were female. Age distributions were also nearly identical, with higher proportions of the deceased aged 25–34 and 35–44 and low proportions in the older and younger age ranges. However, race and ethnicity were not widely available in the JCID and therefore were not included in the comparison. Finally, the majority of the deceased were detained/unconvicted (82% in DCRP data and 89% in JCID data) as opposed to sentenced/convicted in both data sources (17% in DCRP data and 9% in JCID data).

**Table 2:** Demographic Characteristics of the Deceased

		BJS		JCID	
Total		4508		170	
Sex	Male	4122	91%	155	91%
	Female	385	9%	15	9%
Age	≤ 17	48	1%	3	2%
	18–24	827	18%	31	18%
	25–34	1399	31%	46	27%
	35–44	1291	29%	49	29%
	45–54	699	16%	26	15%
	55 ≥	237	5%	3	2%
	Missing	7	0%	12	7%
Legal Status	Convicted / sentenced	715	17%	16	9%
	Unconvicted / detained	3400	82%	151	89%
	Missing	19	0%	3	2%

Jail suicide location statistics were found to be generally similar in rank but divergent in proportion^2^ in the two datasets, with a correlation coefficient of 0.82. Table 3 shows the largest proportion of the reported suicides in both data sources occurred in general housing (47% in DCRP data, 77% in JCID data), followed by segregation units (21.3% in DCRP data and 5.9% in JCID data) and medical units (23.6% in DCRP data and 3.5% in JCID data). A discussion of the potential reasons for these divergences is detailed below.

**Table 3:** Location of Jail Suicides^a2^

	BJS	JCID
General housing	46.9%	77.1%
Segregation unit	21.3%	5.9%
Medical unit	23.6%	3.5%
Mental health unit	1.2%	1.2%
In transit	0.4%	0.0%
Elsewhere	6.6%	1.2%
Missing	0.0%	11.2%

^a^Only the percentages were reported in the official data sets (BJS) and did not include confidence intervals

Again, location by state was generally similar between the two data sources (see Table 4). Interestingly, the same three states (i.e., Florida, Texas and California) ranked in the top three for percent of total suicides for the year 2009 and state-level correlation was found to be 0.82. These similarities continued when the state-level data was aggregated to U.S. Division and U.S. Region, with 0.78 and 0.89 correlations respectively. Of course, a significant factor in these correlations is likely to be the population base of these states; for example, Florida, Texas and California had the highest proportions of suicides and are the most populous in the country, while New England states had lower proportions and have lower populations.

**Table 4:** Division-Level Suicide Proportions^a^

	BJS	JCID
East North Central	14%	14%
East South Central	7%	11%
Mountain	11%	9%
Middle Atlantic	10%	12%
New England	4%	3%
Pacific	15%	8%
South Atlantic	021%	22%
West North Central	7%	11%
West South Central	12%	10%

^a^Only the percentages were reported in the official data sets (BJS)

As indicated in Table 5, facility size is where the two datasets diverged the most. While in official statistics, nearly a third of reported suicides occurred in small facilities (i.e., those under 50 beds (32.5%)) and nearly half in facilities under 100 beds (46.6%); however, the JCID’s data demonstrates a wider distribution of incidents across facility sizes with higher proportions reported in larger facilities between 250 and 1500 beds. It is unclear whether official data was utilizing Average Daily Population (ADP) or Rated Capacity (RC) to determine jail facility size, and both variables were available in the JCID with varying levels of availability (95 jails had ADP while 160 had RC). Therefore, both were included with similar results.

**Table 5:** Facility Size^a^

	JCID – ADP	JCID – RC	BJS^b^
< 50	8%	9%	32%
50–99	11%	10%	14%
100–149	3%	8%	9%
150–249	12%	12%	9%
250–499	20%	15%	10%
500–999	16%	20%	6%
1000–1499	17%	15%	8%
1500–1999	4%	4%	6%
≥ 2000	9%	8%	6%

^a^Only the percentages were reported in the official data sets (BJS)

^b^It was unclear whether BJS used average daily population (ADP) or rated capacity (RC)

## Discussion

While there is an abundance of research on jail suicides, the fact that jail suicide rates continue to rise suggests that we may have become too reliant on using the limited number of variables collected through official data to explain jail suicides (Hayes 2013). The current study demonstrates the potential usefulness of open-source data to examine suicides in jail facilities and, as the demand for evidence-based policy and programming increases in the field of corrections, so too does this opportunity for integrating quality sourced publicly available datasets into current research and discourse.

It is important to note that some information was more readily available via official documentation than in the open-source database. In particular, individual race and ethnicity were widely included in official data, yet were frequently not reported in open-source media and/or news, and, where they were reported, were questionable regarding their reliability. The most comparable variables between the two data sets were found in the individual- and incident-level data consisting of other demographic data (i.e., age, sex, and legal status) and location (i.e., where the incident occurred). While the majority of the official data variables were collected in the open-source data, the latter was found to have significantly more range and diversity in the variables collected on all three levels (individual-, facility-, and incident-level) (see Additional file 1).

Incorporating open-source data such as that contained in the JCID can improve upon the overall understanding of the individual-, incident-, and facility-level characteristics of jail suicides. Expanding upon the current data collection process would enable researchers to create a more robust dataset, enrich the overall quality of the variables collected, and allow for a more nuanced understanding of the correlates of jail suicide. While this data also suggests that the JCID and other open-source data could be used as a check for official data, more research needs to be conducted in order to fully determine how this can be accomplished. In sum, the major findings from this study are that open-source protocol can be used to supplement government data in both meaningful and significant ways.

### Limitations

Despite adding meaning to the jail suicide correlates research, the inclusion of open-source data did not come without several limitations. First, there was missing data in the location field for the JCID and location categories had to be collapsed in the JCID to match with the DCRP location categories. Other categories also differed across data sources and some of the available through the Bureau of Justice Statistics categories were not completed in the JCID (e.g., race in the demographics section).

Second, the reported years differed between the two datasets. For example, in the JCID, researchers are able to examine jail suicides occurring in a single year alone (e.g., 2009), whereas the majority of DCRP tables are aggregated across several years. Presumably, the data is presented in the aggregate for reasons of confidentiality; yet, aggregating data at this level makes it hard to single out year-to-year changes in the potential influential factors leading to jail suicides for any given year (Reilly 2016). Furthermore, the DCRP does not include data on any facility that is classified as a temporary lockup (Noonan 2016; Reilly 2016), and several states fail to submit data on jail deaths to the DCRP because their jails and prisons are part of singular, fully integrated system (i.e., CT, DE, HI, RI, and VT) (Noonan 2016).

The collection of open-source data itself also has some limitations. First, an open-sourced database such as the JCID is potentially biased in that it is based on selective media output, where researchers are unable to control what information is available compared to information that may have been kept out of the public sphere. Second, as this study shows, open-source data is often incomplete. For example, we were only able to identify 170 of the 304 officially reported jail suicides (or 56%) which may have been a result of how the media reports on these incidents (e.g., what they consider newsworthy and whether jail size effects news coverage) or how jails choose to make this data available. Despite these differences however, we were still able to produce meaningful and significant results.

It is important to note that there are other reasons that make it difficult to determine the exact number of suicides that occur within any given correctional facility. First, it is almost impossible to acquire data on issues occurring within correctional facilities that have been classified as a “sensitive issue” (Hayes 1983), and it has been suggested that releasing this type of information may discredit all of the stakeholders involved, which may influence reported suicide numbers (Hampton 2016; Tartaro and Ruddell 2006). Second, unintentional suicides (e.g., the individual was trying to inflict self-harm (cutting), or they accidentally overdosed) could potentially be included in general suicide data or suicides may be misclassified as “accidental” if the stakeholders involved are trying to avoid public and legal repercussions (Hampton 2016; Hayes 1983). Third, the place of occurrence for a successful suicide attempt may not be properly recorded if the victim was being treated, transferred, and removed from the facility immediately following the incident occurrence (Hampton 2016; Hayes 1983). Lastly, Hampton (2016) also noted that not all parties who are allowed to complete DCRP reports have a comprehensive understanding of what constitutes an actual death in custody.

## Conclusion

The two main goals of this study were: (1) to determine if open-source data was able to replicate official national jail suicides prevalence rates and (2) to determine if open-source data was able to increase our overall understanding of the correlates of jail suicides. The results from this study reveal support for the argument in favor of including open-source data in jail suicide research because it not only replicated official national jail suicides prevalence rates but it also afforded researchers a more nuanced understanding of the potential correlates of jail suicides.

For almost every variable measured, open-source data captured as much, if not more of the information presented in official data. Variables not available in official data were identified in the open-source database, allowing for researchers to have a better understanding of the situational characteristics of the event. Uncovering and identifying the potential nuances within jail suicide data may allow researchers to explore different situational factors that may decrease the likelihood of jail suicides. For example, researchers can continue to explore how altering environmental factors can minimize the risk and ability for inmates to commit suicide. Thus, prevention may be directly linked to being able to manipulate environmental factors.

Suicide is a complex phenomenon that is the result of the intersection of a multitude of factors (i.e., psychological, social, biological, environmental, and economic). This study illustrates that correctional suicides are best described as a serious public health issue (McMullan 2011), as suicide remains the leading cause of death for jail inmates in the U.S. (Goss et al. 2002; Hayes 1997; Noonan 2016; Noonan et al. 2015). Therefore, it is not sufficient to continue to rely on national best-practice correctional policy guidelines to deter suicide attempts, as American jails remain highly decentralized. Even though jails today are required to screen detainees during intake for both physical and mental health issues, every jail system adopts and develops its own operating processes (McMullan 2011). Furthermore, it is also not enough for departments/agencies to solely respond to matters surrounding inmate suicide as a result of feared litigation through liability cases, as doing so motivates most facilities to implement policies which require minimal suicide prevention provisions.

Hayes (2013) concludes that correctional suicide rates can only be reduced when these facilities adopt a comprehensive methodology, which has yet to be done systematically within the U.S. This current study attempts to demonstrate the usefulness of considering other methods of data collection (i.e., open-source) to examine suicides in jail facilities. Finally, with the rapid growth of correctional critical incident information reported and stored online, the issue is no longer the lack of data, but how to efficiently and effectively access the abundance of online data for research purposes. Plus, with the advancement in software technology and the advent of web data scraping software it is envisioned that in the not too distant future data obtained from texts from online webpages will be a dominant way of collecting certain jail and prison related data.

## Additional file

Additional file 1: **Figure S1.** Variable Comparison – BJS to JCID. **Figure S2.** JCID Suicide-Specific Variables. (DOCX 17 kb)

## Abbreviations

ACA: American correctional association
ADP: Average daily population
BJS: Bureau of Justice Statistics
DCRP: The U.S. Bureau of Justice Statistics’, Deaths in custody reporting program
JCID: Jail correctional incident database
NDI: National Death Index
RC: Rated capacity

## Footnotes

1 One exception is Frank and Aguirre’s (2013) meta-synthesis, which attempts to qualitatively examine pre-existing research on correctional suicides (see Hayes 1997; Suto and Arnaut 2010).

2 Source of this location data was BJS’s DCRP Table 13: Death location of local jail inmates, by cause of death, 2000–2014.
