# Supplementary material for: The validity of open-source data when assessing jail suicides
Source: Health Justice. 2018 May 9;6:11. doi: 10.1186/s40352-018-0069-2 (PMC5955876; doi:10.1186/s40352-018-0069-2)
Supplement: Supplementary file 1 — Figure S1. Variable Comparison – BJS to JCID. Figure S2. JCID Suicide-Specific Variables. (DOCX 17 kb) [file 40352_2018_69_MOESM1_ESM.docx]

**Additional file 1**

**Figure S1. Variable Comparison – BJS to JCID**

| **Facility-level variables** | |
| --- | --- |
| **BJS - 1** | **JCID - 18** |
| Name/contact information | Name/contact information |
|  | Year of facility data |
|  | Facility-level |
|  | Facility type |
|  | Facility security level |
|  | Construction date |
|  | Renovation date |
|  | Facility capacity |
|  | Facility ADP |
|  | Population over capacity |
|  | Facility gender demographics |
|  | Facility population demographics |
|  | Facility age demographics |
|  | Facility racial demographics |
|  | Cost of care |
|  | Facility number of staff |
|  | ACA accreditation |
|  | General notes |

| **Incident-level variables** | |
| --- | --- |
| **BJS - 10** | **JCID - 22** |
| Month of incident | Month of incident |
| Day of incident | Day of incident |
| Year of incident | Year of incident |
| Incident start time ordinal | Incident start time ordinal |
| Cause of death | Cause of death |
| Pre-existing condition? | Pre-existing condition? |
| Medical examiner/coroner's report? | Medical examiner/coroner’s report? |
| Where death occurred | Where death occurred |
| Where in jail? | Where in jail? |
| Receiving care? | Time incident started (hh:mm) |
|  | Incident day of the week |
|  | Type of incident |
|  | Motive for incident |
|  | # of deaths |
|  | # of injured/wounded |
|  | Property damage |
|  | Type of suicide? (see figure 2) |
|  | Hanging/Suffocation ligature item (see figure 2) |
|  | Hanging anchor-point (see figure 2) |
|  | Policy change? |
|  | Environmental change? |
|  | Disciplinary? |

| **Individual-level variables** | |
| --- | --- |
| **BJS - 7** | **JCID - 24** |
| Name | Name |
| Gender | Gender |
| Race | Race |
| DOB | Age |
| Offenses | Conviction/Committing offense |
| Jail status | Jail status |
| Date of admission | Time in custody prior to incident |
|  | Alias |
|  | Nickname |
|  | Sexual orientation |
|  | Religion |
|  | If other, specify |
|  | Prison gang membership |
|  | Gang-member |
|  | Specify offense |
|  | Prior criminal history |
|  | Prior convictions |
|  | Incident type history |
|  | Violent record |
|  | Sex offense record |
|  | Property offense record |
|  | Catalyst event or incident |
|  | Length of sentence |
|  | Sentence Left |

**Figure S2. JCID Suicide-Specific Variables**

| Type of suicide | Hanging |
| --- | --- |
|  | Knife/cutting instrument |
|  | Intentional drug overdose |
|  | Suffocation |
|  | Foreign bodies ingestion |
|  | Jumping |
|  | Other – Describe |
|  | Not applicable |
|  | Missing |
| Hanging/Suffocation ligature item | Bed sheet |
|  | Shirt |
|  | Pants |
|  | Shoelaces |
|  | Belt |
|  | Socks |
|  | Wire |
|  | Rope |
|  | Plastic bag |
|  | Other |
|  | Not applicable |
|  | Missing |
| Hanging anchor point | Overhead pipes |
|  | Cell bars |
|  | Cell door |
|  | Bunk bed |
|  | Sink/toilet fixture |
|  | Other |
|  | Not applicable |
|  | Missing |
